# Risk factors for mid- and long-term mortality in lung transplant recipients aged 70 years and older

**DOI:** 10.1093/icvts/ivae117

**Published:** 2024-07-01

**Authors:** Yining Pan, Jiang Shi, Xuan Li, Xiaojing Luo, Jiaqin Zhang, Caikang Luo, Yanwei Lin, Fei Huang, Wei He, Xiaoqing Lan, Junjie He, Yu Xu, Jianxing He, Xin Xu

**Affiliations:** First Clinical College, Guangzhou Medical University, Guangzhou, China; Department of Thoracic Surgery and Oncology, The First Affiliated Hospital of Guangzhou Medical University, China State Key Laboratory of Respiratory Disease & National Clinical Research Centre for Respiratory Disease, Guangzhou, China; Department of Organ Transplantation, The First Affiliated Hospital of Guangzhou Medical University, China State Key Laboratory of Respiratory Disease & National Clinical Research Centre for Respiratory Disease, Guangzhou, China; Department of Organ Transplantation, The First Affiliated Hospital of Guangzhou Medical University, China State Key Laboratory of Respiratory Disease & National Clinical Research Centre for Respiratory Disease, Guangzhou, China; Department of Thoracic Surgery and Oncology, The First Affiliated Hospital of Guangzhou Medical University, China State Key Laboratory of Respiratory Disease & National Clinical Research Centre for Respiratory Disease, Guangzhou, China; Department of Thoracic Surgery and Oncology, The First Affiliated Hospital of Guangzhou Medical University, China State Key Laboratory of Respiratory Disease & National Clinical Research Centre for Respiratory Disease, Guangzhou, China; Department of Organ Transplantation, The First Affiliated Hospital of Guangzhou Medical University, China State Key Laboratory of Respiratory Disease & National Clinical Research Centre for Respiratory Disease, Guangzhou, China; Department of Thoracic Surgery and Oncology, The First Affiliated Hospital of Guangzhou Medical University, China State Key Laboratory of Respiratory Disease & National Clinical Research Centre for Respiratory Disease, Guangzhou, China; Department of Organ Transplantation, The First Affiliated Hospital of Guangzhou Medical University, China State Key Laboratory of Respiratory Disease & National Clinical Research Centre for Respiratory Disease, Guangzhou, China; Department of Thoracic Surgery and Oncology, The First Affiliated Hospital of Guangzhou Medical University, China State Key Laboratory of Respiratory Disease & National Clinical Research Centre for Respiratory Disease, Guangzhou, China; Department of Organ Transplantation, The First Affiliated Hospital of Guangzhou Medical University, China State Key Laboratory of Respiratory Disease & National Clinical Research Centre for Respiratory Disease, Guangzhou, China; Department of Thoracic Surgery and Oncology, The First Affiliated Hospital of Guangzhou Medical University, China State Key Laboratory of Respiratory Disease & National Clinical Research Centre for Respiratory Disease, Guangzhou, China; Department of Organ Transplantation, The First Affiliated Hospital of Guangzhou Medical University, China State Key Laboratory of Respiratory Disease & National Clinical Research Centre for Respiratory Disease, Guangzhou, China; Department of Thoracic Surgery and Oncology, The First Affiliated Hospital of Guangzhou Medical University, China State Key Laboratory of Respiratory Disease & National Clinical Research Centre for Respiratory Disease, Guangzhou, China; Department of Thoracic Surgery and Oncology, The First Affiliated Hospital of Guangzhou Medical University, China State Key Laboratory of Respiratory Disease & National Clinical Research Centre for Respiratory Disease, Guangzhou, China; Department of Thoracic Surgery and Oncology, The First Affiliated Hospital of Guangzhou Medical University, China State Key Laboratory of Respiratory Disease & National Clinical Research Centre for Respiratory Disease, Guangzhou, China; Department of Organ Transplantation, The First Affiliated Hospital of Guangzhou Medical University, China State Key Laboratory of Respiratory Disease & National Clinical Research Centre for Respiratory Disease, Guangzhou, China; Department of Thoracic Surgery and Oncology, The First Affiliated Hospital of Guangzhou Medical University, China State Key Laboratory of Respiratory Disease & National Clinical Research Centre for Respiratory Disease, Guangzhou, China; Department of Organ Transplantation, The First Affiliated Hospital of Guangzhou Medical University, China State Key Laboratory of Respiratory Disease & National Clinical Research Centre for Respiratory Disease, Guangzhou, China; Department of Thoracic Surgery and Oncology, The First Affiliated Hospital of Guangzhou Medical University, China State Key Laboratory of Respiratory Disease & National Clinical Research Centre for Respiratory Disease, Guangzhou, China; Department of Organ Transplantation, The First Affiliated Hospital of Guangzhou Medical University, China State Key Laboratory of Respiratory Disease & National Clinical Research Centre for Respiratory Disease, Guangzhou, China

**Keywords:** Lung transplantation, 70 years old, Mortality, Risk factors

## Abstract

**OBJECTIVES:**

With increased lung transplantation in those aged 70 and older, limited literature addresses risk factors affecting their survival. Our study aims to identify independent factors impacting mid- and long-term mortality in this elderly population.

**METHODS:**

This study analyzed lung transplant patients over 70 from May 2005 to December 2022 using United Network for Organ Sharing data. The 3- or 5-year cohort excluded multi-organ, secondary transplantation and loss to follow-up. Univariable Cox analysis was conducted to assess recipient, donor and transplant factors. Factors with a significance level of *P* < 0.2 were subsequently included in a multivariable Cox model to identify correlations with 3- and 5-year mortality in patients aged over 70.

**RESULTS:**

Multivariable analysis has identified key factors affecting 3- and 5-year mortality in elderly lung transplant patients over 70. Common notable factors include recipient total bilirubin, intensive care unit status at the time of transplantation, donor diabetes, Cytomegalovirus (CMV) mismatch and single lung transplantation. Additionally, Hispanic/Latino patients and ischaemia time of the transplant significantly impact the 3-year mortality, while recipient age, diabetes, nitric oxide use before transplantation and creatinine were identified as unique independent risk factors affecting the 5-year morality.

**CONCLUSIONS:**

The study identified several independent risk factors that impact the mid- and long-term survival of lung transplantation for individuals over 70 years. These findings can contribute to the optimization of lung transplant treatment strategies and perioperative management in elderly patients, thereby enhancing the survival rate of this age group.

## Graphical Abstract

**Figure:**
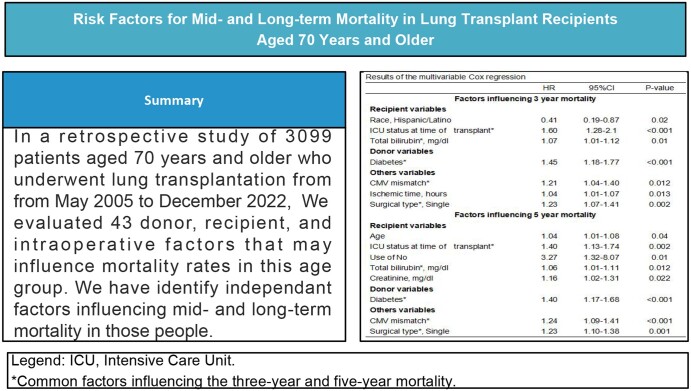

## INTRODUCTION

Lung transplantation is a highly effective treatment for end-stage lung disease, playing a pivotal role in medical advancement. However, with the global increase in the ageing population, older individuals are more commonly affected by end-stage lung disease. According to the latest 2021 document released by International Society for Heart and Lung Transplantation (ISHLT), patients older than 65 years with low physiological reserve and/or other related contraindications are still considered relative contraindications for lung transplants [1]. Nevertheless, the implementation of the lung allocation score (LAS) has led to an increasing number of elderly recipients aged over 70 years [2].

Several studies have been conducted on the survival of lung transplant patients over 70 years old. These studies consistently show that the 3- and 5-year survival rates after lung transplantation in this age group are relatively low [3–6]. This not only indicates a relatively poor prognosis for older recipients after lung transplantation but also suggests that the risk factors they face may differ from those of the general adult population. However, there is currently limited knowledge about the potential risk factors that affect mid- and long-term survival in this specific population. Gaining a deeper understanding of these risk factors can provide more accurate guidance for the clinical treatment of elderly lung transplant patients, evaluate the benefits of lung transplantation for this growing target population, optimize the allocation of transplantation resources, and reduce mid- and long-term mortality after transplantation. Thus, this study aims to comprehensively analyze donor and recipient characteristics, as well as operative factors, to elucidate key determinants influencing mid- and long-term mortality in elderly patients undergoing lung transplantation.

## MATERIALS AND METHODS

### Ethics statement

Before this study was initiated, written approval with waiver of informed consent was obtained from the Institutional Review Board for Guangzhou Medical University Medical Center (ES-2023–226-01). The reason consent was not obtained (Data from United Network for Organ Sharing data (UNOS) public database is anonymized; formal consent from participants was unattainable).

We conducted a retrospective study using data from the Scientific Registry of Transplant Recipients (SRTR) Standard Transplant Analysis and Research files provided by UNOS. The study included patients over 70 years old who underwent lung transplantation in the United States between May 2005 and December 2022. We excluded patients who underwent retransplantation, multi-organ transplantation, and those without survival information, selecting 43 recipient, donor, and intraoperative factors that were biologically plausible and previously reported in the literature.

To address the issue of missing values in the data extracted from UNOS, we employed the Full Conditional Specification method for multiple imputation. Out-of-range continuous variables were truncated to align with clinical norms. Convergence tests were conducted on each dataset, followed by independent statistical analysis of 20 datasets. Finally, Rubin’s principle was applied to summarize the results.

We evaluated the normality of continuous variables through the Kolmogorov–Smirnov test. Normally distributed continuous variables are presented as mean (standard deviation), while non-normally distributed continuous variables are reported as median and interquartile range (IQR). Categorical variables were expressed as counts and percentages. The Kaplan–Meier survival analysis was performed to estimate survival rates.

For factors with *P*-values less than 0.20 in univariable Cox analysis, we incorporated them into a multivariable Cox model through stepwise regression to control for collinearity and optimize the model’s performance. Ultimately, we selected the variables with the greatest impact on mortality as our results. A *P*-value less than 0.05 indicates statistical significance. The data were analyzed using the R statistical software, specifically version 4.3.1 (R Core Team, Austria), and SPSS Version 25 for Windows (SPSS Inc, IBM Corp, Armonk, New York).

## RESULTS

**Figure 1:**
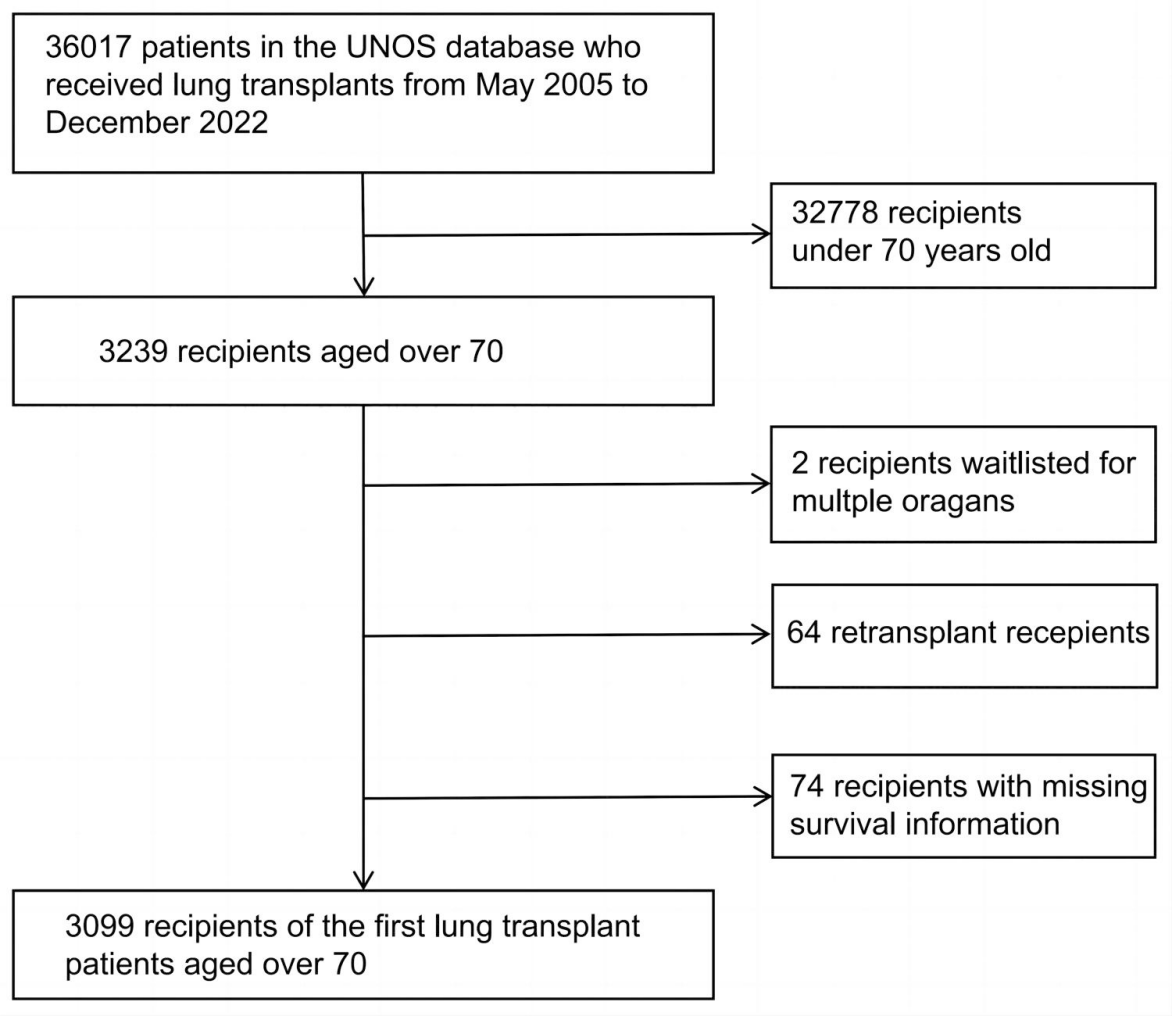
Study cohort.

**Figure 2:**
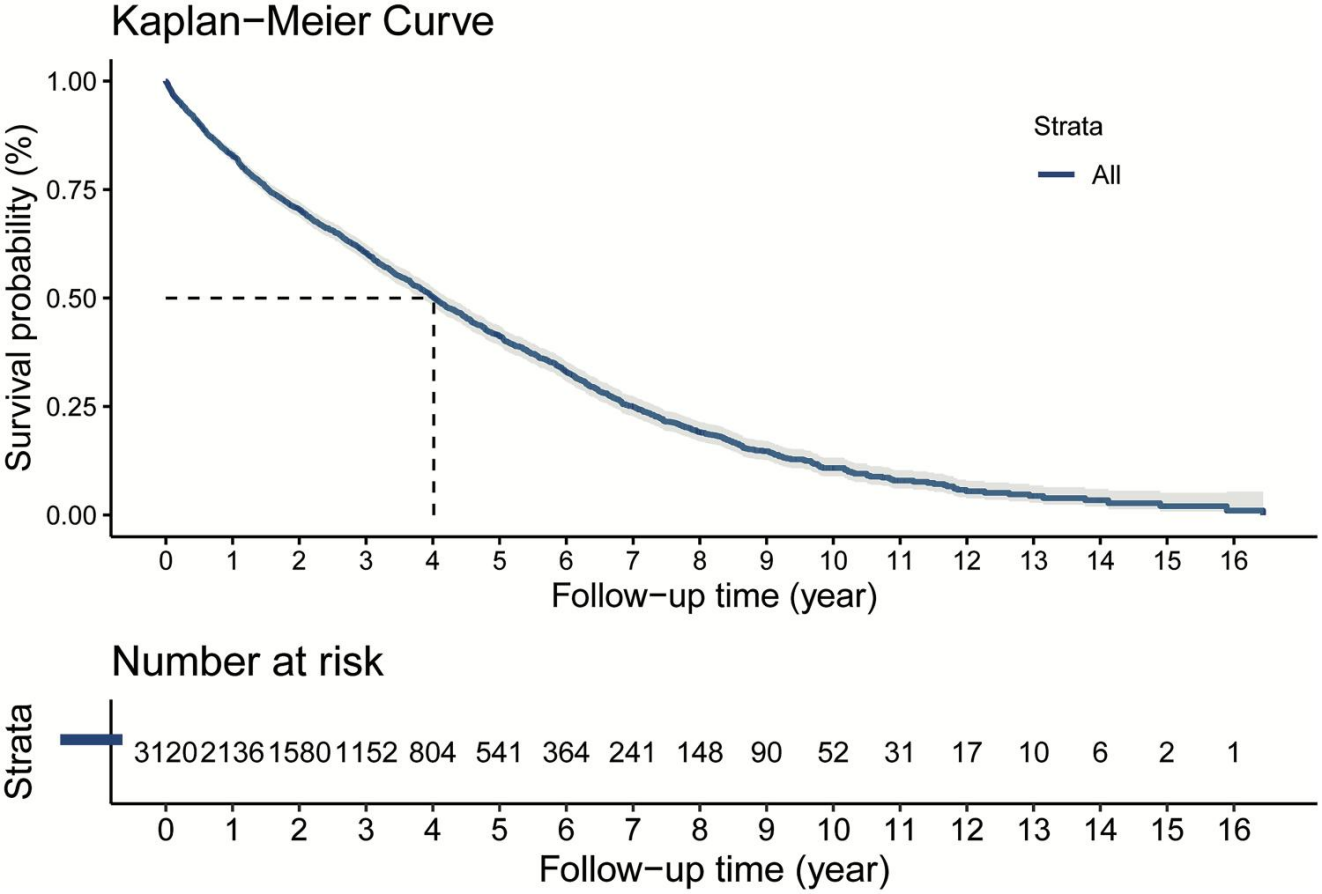
Kaplan–Meier curve of the study cohort.

The study included 3099 lung transplant patients aged over 70 (Fig. 1). In our study cohort, a total of 2316 male patients (74.73%) and 783 female patients (25.27%) underwent lung transplantation surgery. The median age of recipients was 71 years (IQR: 70 to 73). The majority of patients were White (88.42%) and underwent transplantation due to interstitial lung disease (74.57%). Single lung transplantation was performed in the majority of cases (59.73%), with only a fraction of patients being in the intensive care unit (ICU) at the time of transplantation (6.42%). There was a lower prevalence of diabetes among recipients (16.42%) and donors (7.52%). The median ischaemia time for the graft was 4.80 h (IQR: 3.87 to 6.02) (Table 1). The median survival time for these patients was 4.02 years (IQR: 3.82–4.23), with survival rates of 60.4% at 3 years and 41.1% at 5 years (Fig. 2).

**Table 1:** Patient characteristics of the overall population.

	Overall population (*N* = 3099)
Recipient variables	
Male, *n* (%)	2316 (74.73%)
Age, median (IQR)	71 (70, 73)
BMI, mean (SD)	26.1 (3.7)
Race, *n* (%)	
White	2740 (88.42)
Black	91 (2.94)
Hispanic/Latino	168 (5.42)
Asian	83 (2.67)
Blood group, *n* (%)	
A	1276 (41.17)
B	331 (10.69)
O	1372 (44.27)
AB	120 (3.87)
Primary diagnosis, *n* (%)	
Pulmonary vascular disease	49 (1.58)
COPD/emphysema	532 (17.17)
CF/autoimmune disease	32 (1.03)
Interstitial lung disease	2311 (74.57)
Medical condition at transplant	
In ICU, *n* (%)	199 (6.42)
Hospitalized, but not in ICU, *n* (%)	274 (8.84)
Not hospitalized, *n* (%)	2626 (84.74)
Use of ECMO, *n* (%)	28 (0.93)
Use of NO, *n* (%)	7 (0.22)
Use of ventilator, *n* (%)	71 (2.29)
Smoking history, *n* (%)	2099 (67.73)
Tumour, *n* (%)	571 (18.43)
Drug-resistant infection of the lungs, *n* (%)	94 (3.03)
Diabetes, *n* (%)	509 (16.42)
Steroid	1182 (38.14)
Oxygen demand at rest [ml/(kg·min)], median (IQR)	4 (2, 6)
LAS, median (IQR)	41 (35, 50)
FEV1, median (IQR)	53 (38, 67)
FVC, median (IQR)	53 (42, 65)
Total bilirubin, mg/dl, median (IQR)	0.5 (0.3, 0.7)
Creatinine, mg/dl, median (IQR)	0.87 (0.73, 1.01)
Donor variables	
Age, median (IQR)	34 (24, 48)
Male, *n* (%)	1973 (63.67)
Horovitz_index, median (IQR)	4.36 (3.70, 4.96)
BMI, median (IQR)	25.8 (23.6, 28.7)
DCD, *n* (%)	148 (4.78)
Smoking history, *n* (%)	266 (8.58)
Alcohol abuse history, *n* (%)	507 (16.36)
Hypertension, *n* (%)	780 (25.17)
Tumour, *n* (%)	65 (2.1)
Diabetes, *n* (%)	264 (8.52)
MI, *n* (%)	74 (2.39)
Donor infection, *n* (%)	2103 (67.86)
Mechanism of death	
Anoxia, *n* (%)	895 (28.88)
Stroke, *n* (%)	883 (28.49)
Head trauma, *n* (%)	1242 (40.08)
CNS tumour, *n* (%)	17 (0.55)
PCO_2_, median (IQR)	37 (33, 42)
Creatinine, mg/dl, median (IQR)	1.00 (0.77, 1.57)
Total bilirubin, mg/dl, median (IQR)	0.70 (0.50, 1.10)
Mismatch data	
Gender mismatch	
Male-to-Female, *n* (%)	291 (9.39)
Female-to-Male, *n* (%)	634 (20.46)
CMV mismatch	
Donor(+)-to-recipient(−), *n* (%)	789 (25.46)
Donor(−)-to-recipient(+), *n* (%)	615 (19.85)
ABO mismatch, *n* (%)	253 (8.16)
Operative variables	
Ischaemic time, hours, median (IQR)	4.80 (3.87, 6.02)
Surgical type, double, *n* (%)	1245 (40.17)
Centre volume^a^, *n* (%)	
≤25	811 (26.17)
>25, ≤50	660 (21.3)
>50	1628 (52.53)

BMI: body mass index; DCD: donor after cardiac death; ECMO: extracorporeal membrane oxygenation; MI: myocardial infarction; CF: cystic fibrosis; COPD: chronic obstructive pulmonary disease; ICU: intensive care unit; LAS: lung allocation score; FEV1: forced expiratory volume in 1 s; FVC: forced vital capacity.

a The average annual number of lung transplant surgeries performed at a transplant centre.

### Univariable analysis

We conducted an analysis of donor, recipient and intraoperative factors affecting the 3- and 5-year mortality following lung transplantation in elderly patients. Univariable Cox analysis revealed that 10 clinical characteristics significantly influenced the 3-year mortality (*P* < 0.05, Table 2). At the recipient level, those admitted to the ICU at the time of transplantation, patients with pulmonary vascular disease, prior use of ECMO, cholesterol, or nitric oxide (NO), high LAS scores, and high total bilirubin, were significantly associated with mortality rates. Donor comorbidities such as diabetes and hypertension also impacted mortality rates. Further analysis revealed a significant association between CMV mismatch and mortality.

**Table 2:** Univariable and multivariable Cox regression analyses for 3-year mortality

	Univariable model			Multivariable analysis		
	HR	95% CI	*P*-value	HR	95% CI	*P*-value
Recipient variables						
Male	1.11	0.96–1.28	0.16			
Age	1.03	0.99–1.06	0.072			
BMI	1.01	0.99–1.03	0.16			
Race						
White	0.63	0.32–1.27	0.19	0.52	0.26–1.05	0.069
Black	0.64	0.30–1.40	0.27	0.55	0.25–1.20	0.14
Hispanic/Latino	0.49	0.23–1.05	0.065	0.41	0.19–0.87	0.02
Asian	1.04	0.48–2.27	0.91	0.89	0.41–1.94	0.77
Other	1.00	1.00		1.00	1.00	
Blood group						
A	0.77	0.57–1.05	0.094			
B	0.92	0.65–1.29	0.61			
O	0.86	0.63–1.16	0.31			
AB	1.00	1.00				
Primary diagnosis						
Pulmonary vascular disease	1.79	1.09–2.93	0.021			
COPD/emphysema	1.15	0.84–1.57	0.38			
CF/autoimmune disease	1.02	0.48–2.14	0.97			
Interstitial lung disease	1.11	0.88–1.47	0.48			
Other	1.00	1.00				
Medical condition at transplant						
In ICU	1.55	1.23–1.92	<0.001	1.6	1.28–2.1	<0.001
Hospitalized, but not in ICU	1.08	0.87–1.34	0.49	1.01	0.87–1.35	0.47
Not hospitalized	1.00	1.00				
Use of ECMO	2.01	1.12–3.41	0.009			
Use of NO	2.8	1.05–7.47	0.04			
Use of ventilator	1.38	0.98–1.96	0.067			
Smoking history	0.96	0.84–1.09	0.56			
Tumour	1.04	0.89–1.22	0.61			
Drug-resistant infection of the lungs	0.75	0.55–1.04	0.087			
Diabetes	1.13	0.96–1.33	0.13			
Steroid	1.15	1.01–1.29	0.036			
Oxygen demand at rest [ml/(kg·min)]	1.01	1.00–1.02	0.059			
LAS	1.005	1.00–1.01	0.012			
FEV1	0.99	0.99–1.01	0.14			
FVC	1.00	0.99–1.00	0.82			
Total bilirubin (mg/dl)	1.07	1.02–1.13	0.004	1.07	1.01–1.12	0.01
Creatinine (mg/dl)	1.12	0.97–1.30	0.13			
Donor variables						
Age	0.99	0.99–1.00	0.24			
Male	1.00	0.88–1.14	0.98			
horovitz_index	0.99	0.97–1.01	0.61			
BMI	0.99	0.98–1.00	0.27			
DCD	1.25	0.94–1.67	0.13			
Smoking history	1.15	0.93–1.42	0.21			
Alcohol abuse history	1.09	0.92–1.29	0.32			
Hypertension	1.18	1.03–1.36	0.016			
Tumour	1.19	0.80–1.78	0.38			
Diabetes	1.44	1.18–1.76	<0.001	1.45	1.18–1.77	<0.001
MI	1.27	0.85–1.88	0.24			
Donor infection	1.01	0.88–1.15	0.92			
Mechanism of death						
Anoxia	1.45	0.89–2.51	0.13			
Stroke	1.55	0.92–2.60	0.10			
Head trauma	1.44	0.86–2.42	0.16			
CNS tumour	2.08	0.88–4.90	0.095			
PCO_2_	0.99	0.99–1.01	0.84			
Creatinine (mg/dl)	1.00	0.97–1.04	0.84			
Total bilirubin (mg/dl)	0.945	0.89–1.00	0.069			
Mismatch data						
Gender mismatch						
Male-to-female	1.05	0.85–1.29	0.67			
Female-to-male	1.07	0.92–1.24	0.41			
CMV mismatch						
Donor(+)-to-recipient(−)	1.2	1.04–1.39	0.015	1.21	1.04–1.40	0.012
Donor(−)-to-recipient(+)	1.03	0.87–1.22	0.71	1.04	0.88–1.22	0.64
ABO mismatch	1.13	0.91–1.41	0.26			
Operative variables						
Ischaemic time (hours)	1.03	0.99–1.06	0.059	1.04	1.01–1.07	0.013
Surgical type, single	0.89	0.78–1.01	0.069	1.23	1.07–1.41	0.002
Centre volume^a^						
≤25	1.13	0.98–1.31	0.10			
>25, ≤50	0.97	0.82–1.14	0.67			
>50	1.00	1.00				

BMI: body mass index; CF: cystic fibrosis; COPD: chronic obstructive pulmonary disease; DCD: donor after cardiac death; ECMO: extracorporeal membrane oxygenation; FEV1: forced expiratory volume in 1 s; FVC: forced vital capacity; HR: hazard ratio; ICU: intensive care unit; LAS: lung allocation score; MI: myocardial infarction.

a The average annual number of lung transplant surgeries performed at a transplant centre.

Thirteen factors significantly impacted 5-year mortality (*P* < 0.05, Table 3). We observed that more recipient characteristics influenced 5-year mortality compared to 3-year mortality. In addition to diagnoses of pulmonary vascular disease, being in the ICU at the time of transplantation, prior use of cholesterol, NO, and high total bilirubin, factors such as blood type A, prior use of mechanical ventilation, diabetes, and elevated creatinine level were also closely associated with mortality rates. Furthermore, single lung transplantation affects 5-year mortality rates, while other factors, such as donor diabetes, donor hypertension and CMV mismatch, also impact 5-year survival rates.

**Table 3:** Univariable and multivariable Cox regression analyses for 5-year mortality

	Univariable model			Multivariable model		
	HR	95% CI	*P*-value	HR	95% CI	*P*-value
Recipient variables						
Male	1.13	0.99–1.28	0.067			
Age	1.04	1.02–1.07	0.001	1.04	1.01–1.08	0.04
BMI	1.01	0.99–1.03	0.13			
Race						
White	0.71	0.37–1.36	0.3			
Black	0.85	0.42–1.74	0.66			
Hispanic/Latino	0.59	0.29–1.21	0.15			
Asian	0.93	0.45–1.95	0.86			
Other	1.00	1.00				
Blood group						
A	0.73	0.56–0.95	0.02			
B	0.82	0.61–1.01	0.18			
O	0.79	0.62–1.04	0.09			
AB	1.00	1.00				
Primary diagnosis						
Pulmonary vascular disease	1.61	1.01–2.55	0.04			
COPD/emphysema	1.12	0.85–1.47	0.43			
CF/autoimmune disease	1.07	0.55–2.08	0.85			
Interstitial lung disease	1.11	0.87–1.43	0.41			
Other	1.00	1.00				
Medical condition at transplant						
In ICU	1.33	1.08–1.64	0.008	1.40	1.13–1.74	0.002
Hospitalized, but not in ICU	1.09	0.90–1.32	0.37	1.13	0.94–1.37	0.2
Not hospitalized	1.00	1.00		1.00	1.00	
Use of ECMO	1.51	0.89–2.56	0.12			
Use of NO	3.4	1.41–8.19	0.006	3.27	1.32–8.07	0.01
Use of ventilator	1.34	1.01–1.85	0.041			
Smoking history	1.00	0.89–1.13	0.97			
Tumour	1.06	0.92–1.22	0.43			
Drug-resistant infection of the lungs	0.93	0.49–1.77	0.82			
Diabetes	1.15	1.01–1.33	0.049	1.16	1.01–1.34	0.038
Steroid	1.09	0.98–1.22	0.11			
Oxygen demand at rest, ml/(kg·min)	1.00	0.99–1.01	0.3			
LAS	1.00	0.99–1.01	0.099			
FEV1	0.99	0.99–1	0.56			
FVC	1.00	0.99–1	0.97			
Total bilirubin, mg/dl	1.06	1.01–1.1	0.015	1.06	1.01–1.11	0.012
Creatinine, mg/dl	1.15	1.01–1.31	0.037	1.16	1.02–1.31	0.022
Donor variables						
Age	1.00	0.99–1.00	0.25			
Male	1.03	0.92–1.15	0.6			
Horovitz index	0.99	0.99–1	0.49			
BMI	1.00	0.99–1.01	0.82			
DCD	1.22	0.93–1.61	0.15			
Smoking history	1.15	0.96–1.39	0.13			
Alcohol abuse history	1.03	0.89–1.20	0.69			
Hypertension	1.21	1.07–1.37	0.002			
Tumour	1.03	0.71–1.49	0.87			
Diabetes	1.42	1.18–1.70	<0.001	1.40	1.17–1.68	<0.001
MI	1.25	0.86–1.78	0.22			
Donor infection	1.03	0.92–1.16	0.62			
Mechanism of death						
Anoxia	1.26	0.84–1.91	0.27			
Stroke	1.30	0.86–1.96	0.21			
Head trauma	1.21	0.81–1.82	0.36			
CNS tumour	1.35	0.61–2.99	0.47			
PCO_2_	0.99	0.99–1	0.97			
Creatinine, mg/dl	1.01	0.98–1.04	0.42			
Total bilirubin, mg/dl	0.98	0.94–1.02	0.33			
Mismatch data						
Gender mismatch						
Male-to-female	1.00	0.83–1.21	0.99			
Female-to-Male	1.06	0.93–1.21	0.36			
CMV mismatch						
Donor(+)-to-recipient(−)	1.21	1.06–1.37	0.005	1.24	1.09–1.41	<0.001
Donor(−)-to-recipient(+)	1.03	0.89–1.19	0.69	1.03	0.89–1.19	0.677
ABO mismatch	1.04	0.86–1.23	0.66			
Operative variables						
Ischaemic time, hours	1.01	0.98–1.04	0.49			
Surgical type, single	0.82	0.73–0.92	<0.001	1.23	1.10–1.38	0.001
Centre volume^a^						
≤25	1.05	0.93–1.20	0.42			
>25, ≤50	0.88	0.77–1.02	0.098			
>50	1.00	1.00				

BMI: body mass index; CF: cystic fibrosis; COPD: chronic obstructive pulmonary disease; DCD: donor after cardiac death; ECMO: extracorporeal membrane oxygenation; FEV1: forced expiratory volume in 1 s; FVC: forced vital capacity; HR: hazard ratio; ICU: intensive care unit; LAS: lung allocation score; MI: myocardial infarction.

a The average annual number of lung transplant surgeries performed at a transplant centre.

### Multivariable analysis

In the 3-year survival cohort, we utilized forward stepwise regression to incorporate variables with *P* < 0.2 from the univariable Cox analysis into the multivariable Cox analysis. Through this stepwise regression process, In our final optimized multivariable Cox model, 7 factors were included, Hispanic/Latino ethnicity (Hazard ratio [HR]: 0.41; IQR: 0.19–0.87), ICU status at the time of transplantation (HR: 1.6; IQR: 1.28–2.1), total bilirubin (HR: 1.07; IQR: 1.01–1.12), donor diabetes (HR: 1.45; IQR: 1.18–1.77), CMV mismatch (HR: 1.21; IQR: 1.04–1.40), ischaemia time (HR: 1.04; IQR: 1.01–1.07) and single lung transplantation (HR: 1.23; IQR: 1.07–1.41).

For the 5-year survival cohort, we employed stepwise regression and identified recipient age (HR: 1.04; IQR: 1.01–1.08), ICU status at the time of transplantation (HR: 1.4; IQR: 1.13–1.74), pretransplant use of NO (HR: 3.27; IQR: 1.32–8.07), diabetes (HR: 1.16; IQR: 1.01–1.34), total bilirubin (HR: 1.06; IQR: 1.01–1.11), creatinine (HR: 1.16; IQR: 1.03–1.31), donor diabetes (HR: 1.4; IQR: 1.17–1.68), CMV mismatch (HR: 1.24; IQR: 1.09–1.41) and single lung transplantation (HR: 1.23; IQR: 1.10–1.38) as independent risk factors affecting the 5-year mortality rate.

## DISCUSSION

Since the post-LAS era, there has been a notable rise in lung transplants performed on individuals aged 70 and above. Despite this increase, there exists limited understanding of the factors influencing mortality within this specific demographic. The significance of this study lies in 3 main aspects: (i) The physical condition of elderly patients differs from that of adult patients, and even among different age groups of elderly individuals. The risk factors that apply to adults or other age groups of elderly individuals may not necessarily apply to those aged 70 and above. (ii) There is currently a lack of comprehensive research on the risk factors associated with lung transplant survival in this specific population. Previous studies on post-transplant outcomes in the elderly have examined fewer risk factors, potentially affecting the results of multivariable analysis. (iii) Limited exploration of separating factors influencing mid- and long-term survival rates were conducted [7–9]. Therefore, our study aims to identify the independent risk factors impacting the 3- and 5-year mortality of elderly patients. This approach offers several advantages. Firstly, it allows us to identify stage-specific risk factors and reduce confounding variables. Secondly, it enables us to provide more targeted interventions. In this study, we have compiled a total of 43 factors, which were both biologically plausible and previously reported in the literature, to comprehensively explore the factors that affect mortality outcomes in patients aged over 70.

In our multivariable model, several common risk factors were found to affect the mid- and long-term survival of individuals aged over 70 years, including ICU status at the time of transplantation, total bilirubin, donor diabetes and CMV mismatch. Ischaemia time is an independent risk factor affecting the 3-year mortality rate, while diabetes is an independent factor affecting the 5-year mortality rate. These risk factors were similar to those observed in previous studies on adult lung transplantation [9–11]. Mosher and colleagues [7] conducted a retrospective cohort study in 2020 using the SRTR data for individuals aged 65 years and above. Their study found that creatinine significantly influenced the elderly mortality, which is similar to our results. However, we discovered that the recipient creatinine level was identified as an independent risk factor affecting the 5-year mortality in this population, but it did not have an impact on the 3-year mortality. Our approach may reflect that the impact of creatinine on survival rates becomes progressively evident over time or is associated with complications occurring later in the course, thus providing more targeted clinical guidance.

NO is a vasodilator that has been extensively studied for its mechanisms, and it has been demonstrated in numerous studies that its use during or after lung transplantation can improve postoperative survival rates for patients [12], But previous studies have not rigorously demonstrated beneficial effects of pretransplant prophylactic inhaled NO on lung transplantation [13]. We speculate that this discrepancy may be related to the poorer health status of patients requiring NO before transplantation, rather than due to prophylaxis.

In previous studies involving elderly lung transplant recipients, the influence of race which appeared to be insignificant was not emphasized. However, our findings indicate that race certainly impacts the mortality of elderly patients. Specifically, we observed that Hispanic and Latino individuals exhibit a significantly lower 3-year mortality compared to other ethnic groups. We hypothesize that this difference may be attributed to genetic factors or influenced by unique lifestyle and social-psychological factors within their ethnicity. A recent population-based study using the UNOS database also demonstrated similar results [14]. Their findings indicated a reduced 5-year mortality risk among Latino individuals. However, race did not significantly affect the 5-year mortality rate in our study.

Controversy has surrounded lung transplant surgery for individuals over the elderly. Early studies primarily focused on conducting single lung transplantation in older patients [15]. However, recent studies suggest that double lung transplantation may offer a similar or higher survival rate for elderly individuals [3]. Our study found that single lung transplantation is closely associated with the mid- to long-term mortality in elderly patients. Therefore, in the early years, medical teams may have considered single lung transplantation to be a relatively conservative option, with lower surgical risks, suitable for older patients. However, as time has passed and medical technology has advanced, we found that single lung transplantation is not a suitable option for elderly individuals compared to double lung transplantation surgery.

Our study has several limitations. Firstly, our research is based on the LAS system, which is currently the most widely used. The selected data spans from May 2005 to the end of December 2022, as this is the cutoff point of UNOS data. Therefore, the findings of this study may require further exploration in future research to assess the effectiveness of the composite allocation score (CAS) system that has implemented in March 2023. Secondly, it is worth mentioning that the data analyzed in our study are exclusively from centres in the United States, potentially limiting the generalizability of our findings to other regions worldwide. Secondly, it is important to note that our study is a broad survey conducted on patients over 70 years old. What’s more, the influencing factors we explore may not necessarily vary across specific age groups. Lastly, while we have determined the direct relationship between continuous variable risk factors and survival rate, we have not identified the specific cutoff points that may impact the medium and long-term survival.

## CONCLUSION

Our study has significant clinical implications for the field of lung transplantation in elderly individuals aged 70 years and above. We offer valuable insights for clinicians and transplant teams, underscore the importance of considering patient demographics and transplant procedures. In line with our expectation, the factors influencing survival after lung transplantation in adults may not necessarily apply to older patients. This discrepancy may be attributed to age-related differences in body structure, particularly in the elderly population, which become more pronounced with increasing age. Therefore, future research could delve deeper into exploring the factors contributing to these age-related differences and further stratify the study population based on age.

## Data Availability

The data underlying this article are available in the article.

## ABBREVIATIONS

HR: Hazard ratios
ICU: Intensive care unit
ISHLT: International Society for Heart and Lung Transplantation
IQR: Interquartile range
LAS: Lung allocation score
NO: Nitric oxide
SRTR: Scientific Registry of Transplant Recipients
UNOS: United Network for Organ Sharing data
